# Personalized risk prediction of mortality and rehospitalization for heart failure in patients undergoing mitral valve repair surgery

**DOI:** 10.3389/fcvm.2024.1470987

**Published:** 2024-11-01

**Authors:** Ning Zhou, Kui Zhang, Bokang Qiao, Cong Chen, Xiaobo Guo, Wei Fu, Jubing Zheng, Jie Du, Ran Dong

**Affiliations:** ^1^ Coronary Artery Disease Surgical Center, Beijing Anzhen Hospital, Capital Medical University, Chaoyang District, China; ^2^ Department of Cardiac Surgery, Beijing Anzhen Hopital, Capital Medical University, Beijing, China; ^3^ Precision Medicine Center, Beijing Institute of Heart, Lung and Blood Vessel Diseases, Beijing Anzhen Hospital, Capital Medical University, Beijing, China

**Keywords:** personalized risk prediction, rehospitalization for heart failure, mortality, mitral valve repair (MV repair), machine learning (ML)

## Abstract

**Background:**

Accurately assessing the postoperative mortality and rehospitalization for heart failure risks in patients undergoing mitral valve repair surgery is of significant importance for individualized medical strategies.

**Objective:**

We sought to develop and validate a risk assessment system for the prediction of mortality and rehospitalization for heart failure.

**Methods:**

Personalized risk prediction system of mortality and rehospitalization for heart failure was developed. For developing a prediction system with death as the outcome, there were 965 patients (70%) and 413 patients (30%) were included in the the derivation cohort and the validation cohort. For developing a prediction system with rehospitalization for heart failure as the outcome, there were 927 patients (70%) and 398 patients (30%) were included in the derivation cohort and the validation cohort. There were 42 routine clinical variables used to develop the models. The performance evaluation of the model is based on the area under the curve (AUC). Evaluate the improvement with Euro Score II according to NRI and IDI net reclassification improvement (NRI) and integrated discrimination improvement (IDI).

**Results:**

The median follow-up time was 685 days, the incidence of death was 3.85% (*n* = 53), and the incidence of rehospitalization for heart failure was 10.01% (*n* = 138). The AUC values of the mortality prediction model in the derivation and validation cohorts were 0.825 (0.764–0.886) and 0.808 (0.699–0.917), respectively. The AUC values of the rehospitalization for heart failure prediction model in the derivation and validation cohorts were 0.794 (0.756–0.832) and 0.812 (0.758–0.866), respectively. NRI and IDI showed that the mortality prediction model exhibited superior performance than the Euro Score II. The mortality and rehospitalization for heart failure risk prediction models effectively stratified patients into different risk subgroups.

**Conclusion:**

The developed and validated models exhibit satisfactory performance in prediction of all-cause mortality and rehospitalization for heart failure after mitral valve repair surgery.

**Clinical Trial Registration:**

http://www.clinicaltrials.gov, Unique identifier: (NCT05141292).

## Introduction

Mitral regurgitation increases the left-ventricular volume load of the heart, which is closely related to decreased quality of life, increased hospitalization rates for heart failure, and shortened survival (1, 2). The management guidelines for valvular heart disease recommend surgical mitral valve repair to improve clinical prognosis for patients with moderate to severe mitral regurgitation with heart failure (3). However, despite correcting hemodynamics through surgery, some patients still suffer from heart failure or even death (4). Perioperative adverse events are only a part of the overall prognosis of patients, postoperative survival rate and and whether patients have heart failure symptoms or even readmission are the important indicators for evaluating the prognosis of patients (5). Therefore, personalized prognostic assessment of patients after mitral valve repair surgery and identifying high-risk patients will help with personalized clinical management, but this clinical need has not yet been met.

Accurately assessing the postoperative mortality risk and cardiac function improvement of patients is of great significance for personalized medical treatment strategies (3). The existing risk prediction models are mainly based on preoperative indicators and surgical factors for predicting perioperative risks. However, given that adverse events occurring during the perioperative period only account for a small part of the overall prognosis, the ability to evaluate residual postoperative risks is insufficient (6). Guideline for the management of patients with valvular heart disease recommend that in addition to the patient's clinical characteristics and surgical procedures, laboratory indicators reflecting the pathophysiological status and echocardiography indicators reflecting the cardiac structure and load should be used to assist in the assessment of individual prognosis (3, 7).

The current risk prediction tools use traditional stepwise regression modeling techniques, which are constrained by the normality of variable distribution, missing values, and nonlinear relationships between variables. Machine learning methods can capture high-dimensional and nonlinear relationships between a large number of clinical features to overcome these limitations (8). This method has been proven effective in the medical application of cardiovascular diseases (9). Therefore, we attempted to develop a personalized risk prediction system that combines clinical data, imaging, surgical procedures and clinical laboratory indicators to evaluate the risk of death and readmission for heart failure after mitral valve repair treatment.

## Methods

### Data sources and study population

The research subjects were patients undergoing mitral valve repair surgery at Beijing Anzhen Hospital from March 2016 to January 2020. The study was approved by the local ethics committee, and the research protocol met the requirements of the Declaration of Helsinki. All participants signed a written informed consent form (NCT05141292).

Each patient was diagnosed through echocardiography and underwent surgery in accordance with the widely accepted treatment indications. We included patients who underwent mitral valve repair surgery and excluded patients who were unable to obtain medical records, under 18 years of age, lost to follow-up, or had more than 50% missing research data (Detailed in the Supplementary Methods S1).

**Figure 1:**
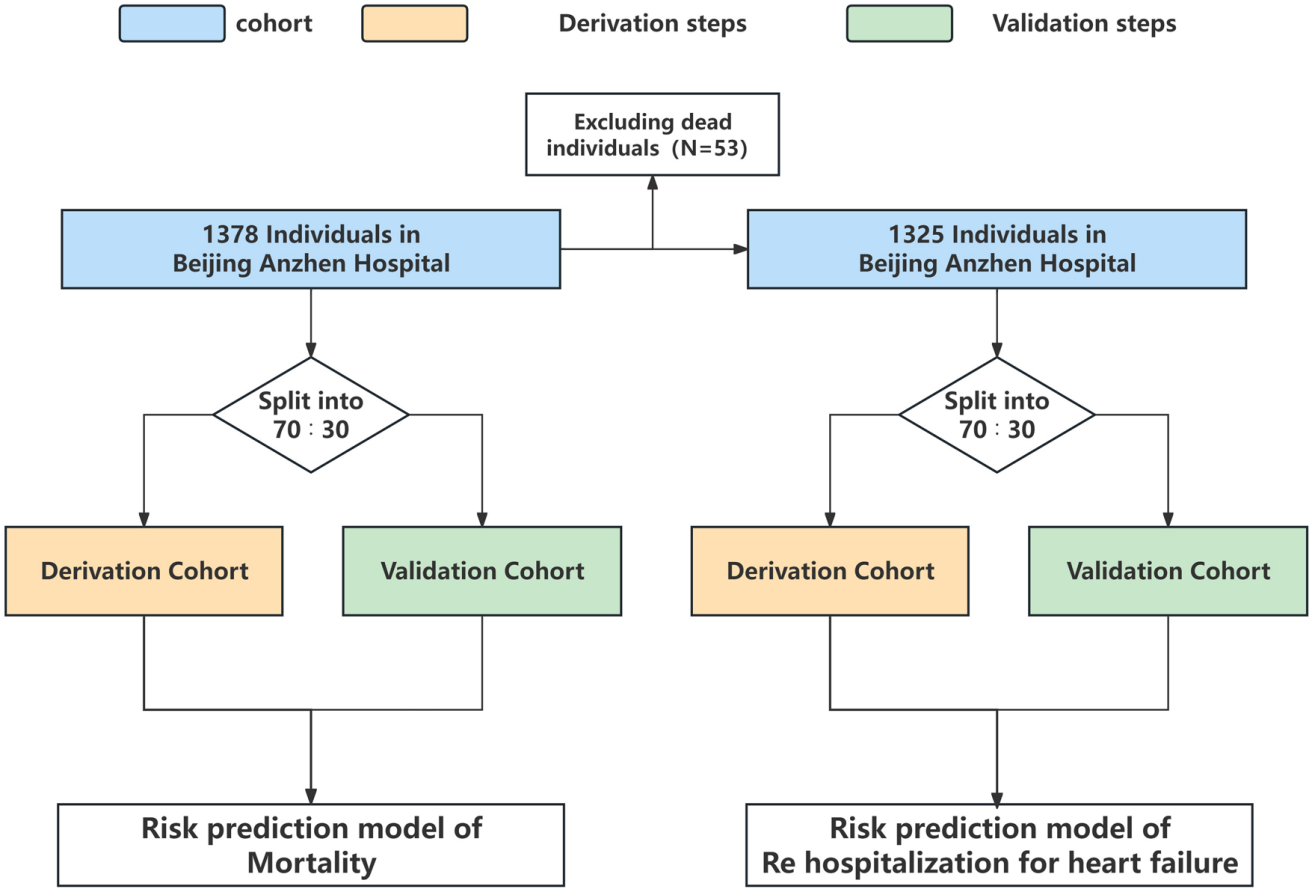
Analysis overview for identifying best-performing risk prediction model.

For developing a prediction model with death as the outcome, 965 patients (70%) were included in the derivation cohort, while 413 patients (30%) were included in the validation cohort. For developing a prediction model with rehospitalization for heart failure as the outcome, the derivation cohort included 927 patients (70%) and the validation cohort included 398 patients (30%) (Figure 1).

### Outcomes

The outcomes were all-cause mortality and rehospitalization for heart failure. We collected patient prognostic information from medical records and telephone follow-up. The follow-up period ranged from discharge after mitral valve repair surgery to 1,702 days, with a median follow-up time of 685 days.

### Feature selection, sample size and missing values

The clinical data was collected from patients’ electronic medical records. Before conducting data analysis, we first implemented strict data quality control. The candidate variables include clinical features, imaging, surgery, and laboratory variables of the patient, totaling 42 variables. Both ultrasound and laboratory indicators were obtained from the last test before discharge. Each variable is detailed in the Supplementary Methods S1.

We utilized R software to estimate the required sample sizes for our predictive models. For the mortality prediction model, we assumed that the model's performance could reach an area under the curve (AUC) of 0.8, with an event occurrence rate of 0.05 and nine predictor variables in the risk model. The minimum required sample size was calculated to be 1,259 cases. For the rehospitalization for heart failure risk prediction model, we similarly assumed a model performance reaching an AUC of 0.8, with an event occurrence rate of 0.10 and nine predictor variables as well. The minimum required sample size for this model was determined to be 686 cases.

Supplementary Tables S1, S2 display the missing values. We used multiple interpolation methods to deal with missing values, we utilized 10 datasets for conducting multiple imputation inference. The risk prediction system was developed and validated for each interpolated dataset. The average values from the models were utilized for performance evaluation and validation.

### Statistical methods

We respectively used XG Boost and traditional logistic regression to develop model and compare the efficiency of the models (variable entry *P* < 0.05, variable removal *P* > 0.05).

The model discrimination is estimated by calculating the area under the curve (AUC) using the receiver operating characteristic curve. The Hosmer-Lemeshow test is employed to assess the goodness of fit of the model by evaluating the discrepancies between predicted values and actual outcomes. Comparisons with the traditional models were based on determining net reclassification improvement (NRI) and integrated discrimination improvement (IDI). In order to compare the mortality risk prediction model with existing risk prediction strategies (Euro Score II) (10). Based on the follow-up outcomes and time, the Kaplan-Meier estimate was applied to calculate absolute risk by using the quartiles of the risk model scores. In addition, nomograms were developed to individualize the prediction of all-cause mortality and rehospitalization for heart failure.

### Statistical analysis

Most analyses were performed using Stata 17.0 (Stata Corp, College Station, Texas, USA) and the R software package (R Foundation for Statistical Computing, Vienna, Austria). Statistical significance was defined by a two-tailed *P*-value <0.05.

## Results

A total of 1,378 patients were included in this study. During the median follow-up time of 685 days, the overall incidence of death was 3.85% (*n* = 53), and the overall incidence of rehospitalization for heart failure was 10.01% (*n* = 138).

Excluding hyperlipidemia, history of central nervous system diseases, and the level of creatine kinase MB (CKMB), no significant differences were observed between the derivation cohort and the validation cohort for developing the prediction model of mortality (Table 1). No significant differences were observed between the derivation cohort and the validation cohort for constructing the prediction model of rehospitalization for heart failure, except for the level of N-terminal pro-brain natriuretic peptide (NT-Pro BNP) (Supplementary Table S3).

**Table 1:** Baseline characteristics of the included cohorts (*n* = 1,378)*.

Variables	Derivation cohort (*n* = 965)	Validation cohort (*n* = 413)	*p* value
Demographic variables			
Women (*N*, %)	549 (56.89)	235 (56.90)	0.99
Age (Median ± SD)	56.71 ± 11.58	55.89 ± 11.68	0.23
Euro score II	2.90 ± 2.64	2.96 ± 2.69	0.68
Clinical variables			
Smoking (*N*, %)	223 (23.11)	113 (27.36)	0.09
Drinking (*N*, %)	163 (16.89)	71 (17.19)	0.89
Hypertension (*N*, %)	316 (32.75)	139 (33.66)	0.74
Diabetes (*N*, %)	84 (8.70)	36 (8.72)	0.99
Hyperlipidemia (*N*, %)	101 (10.47)	26 (6.30)	0.01
CAD (*N*, %)	170 (17.62)	66 (15.98)	0.46
Syncope (*N*, %)	17 (1.76)	10 (2.42)	0.42
AF (*N*, %)	357 (36.99)	144 (34.87)	0.45
Pre-MI (*N*, %)	28 (2.9)	5 (1.21)	0.06
Pre-surgery (*N*, %)	37 (3.83)	16 (3.87)	0.97
Pre-valve surgery (*N*, %)	7 (0.73)	5 (1.21)	0.37
Renal insufficiency (*N*, %)	18 (1.87)	5 (1.21)	0.39
Infectious endocarditis (*N*, %)	21 (2.18)	7 (1.69)	0.56
Central nervous (*N*, %)	72 (7.46)	19 (4.60)	0.05
Lung disease (*N*, %)	25 (2.59)	7 (1.69)	0.31
Peripheral vd (*N*, %)	9 (0.93)	5 (1.21)	0.64
Imaging variables			
LA (Median ± SD)	38.72 ± 6.85	38.54 ± 6.40	0.67
VST (Median ± SD)	10.09 ± 1.85	10.03 ± 1.71	0.67
LVEDD (Median ± SD)	47.69 ± 6.04	47.67 ± 5.85	0.94
LVESD (Median ± SD)	32.94 ± 6.21	32.90 ± 6.01	0.91
Lv thickness (Median ± SD)	9.74 ± 1.50	9.72 ± 1.38	0.83
LVEF (Median ± SD)	57.16 ± 8.09	57.67 ± 7.31	0.30
SPAP (Median ± SD)	23.58 ± 8.00	23.61 ± 7.80	0.96
TAPSE (Median ± SD)	19.43 ± 0.09	19.27 ± 0.14	0.35
Laboratory variables			
NT-Pro BNP (Median ± SD)	1090.59 ± 2078.55	1147.50 ± 3173.83	0.74
CKMB (Median ± SD)	52.51 ± 41.08	58.53 ± 47.27	0.02
TNI (Median ± SD)	5.15 ± 7.75	4.98 ± 5.68	0.69
CRP (Median ± SD)	25.48 ± 42.84	29.59 ± 46.07	0.16
Cr (Median ± SD)	72.09 ± 30.63	73.45 ± 37.84	0.49
Hb (Median ± SD)	102.83 ± 21.36	104.37 ± 20.49	0.23
Lym (Median ± SD)	1.42 ± 1.19	1.35 ± 0.95	0.30
Neu (Median ± SD)	10.24 ± 6.45	9.71 ± 4.35	0.13
PLT (Median ± SD)	150.49 ± 70.32	152.47 ± 76.33	0.65
Surgical variables			
Combined aortic surgery (*N*, %)	28 (2.9)	16 (3.87)	0.35
Combined avr (*N*, %)	116 (12.02)	55 (13.32)	0.50
Combined tvp (*N*, %)	545 (56.48)	229 (55.45)	0.72
Combined cabg (*N*, %)	131 (13.58)	53 (12.83)	0.71
Combined ra (*N*, %)	285 (29.53)	126 (30.51)	0.72
Combined asd (*N*, %)	19 (1.97)	10 (2.42)	0.59
Combined vsd (*N*, %)	2 (0.21)	3 (0.73)	0.14
Aortic cross clamp time (Median ± SD)	94.08 ± 40.49	97.67 ± 35.14	0.19
Follow-up events			
Days (*N*, %)	684.84 ± 140.25	684.26 ± 253.45	0.97
Death (*N*, %)	37 (3.42)	16 (3.87)	0.97
Rehospitalization for heart failure (*N*, %)	91 (9.81)	46 (11.59)	0.33

CAD, coronary heart disease; AF, atrial fibrillation; Pre-MI, previous myocardial infarction; Peripheral vd, peripheral vascular disease; Pre-surgery, previous surgery; Pre-valve surg, previous valve surgery; Central nervous, previous central nervous system disease; LA, left atrial size (mm); VST, ventricular septal thickness (mm); LVEDD, left-ventricular end-diastolic volume (mm); LVESD, left-ventricular end-systolic diameter (mm); Lv thickness, left-ventricular wall thickness (mm); LVEF, left-ventricular ejection fraction (%); SPAP, systolic pulmonary artery pressure (mmHg); TAPSE, tricuspid annular plane systolic excusion (mm); NT-Pro BNP, N-terminal pro-brain natriuretic peptide (pg/ml); CKMB (ng/ml), creatine kinase MB; TNI, cardiac troponin I (ng/ml); CRP, C-reactive protein (mg/L); Cr, serum creatinine (umol/L); Hb, hemoglobin (g/L); Lym, lymphocyte count (*10^−9^/L); Neu, neutrophil count (*10^−9^/L); PLT, platelet count (*10^−9^/L); avr, aortic valve surgery; tvp, tricuspid valve repair surgery; ra, radiofrequency ablation; cabg, coronary artery bypass grafting; asd, atrial septal repair; vsd, ventricular septal repair.

* For continuous variables, non-normally distributed variables are expressed as median [interquartile ranges (IQRs)] and normally distributed variables are expressed as means [standard deviation (SD)]. Categorical variables are expressed as *N* (%). *P* values less than 0.05 were considered statistically significant.

**Figure 2:**
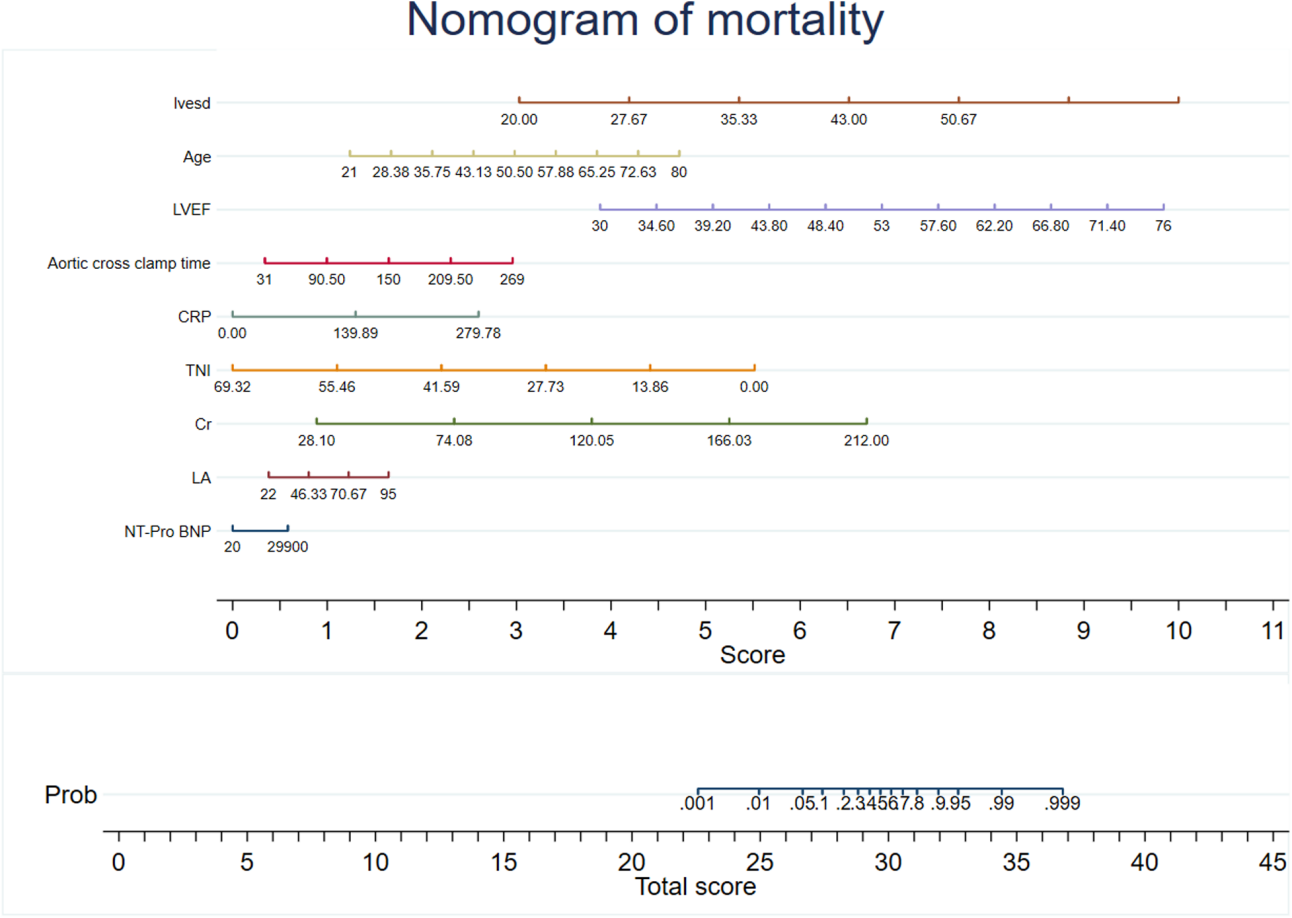
Nomogram of mortality. The nomogram was constructed based on the regression coefficient in the model.

**Figure 3:**
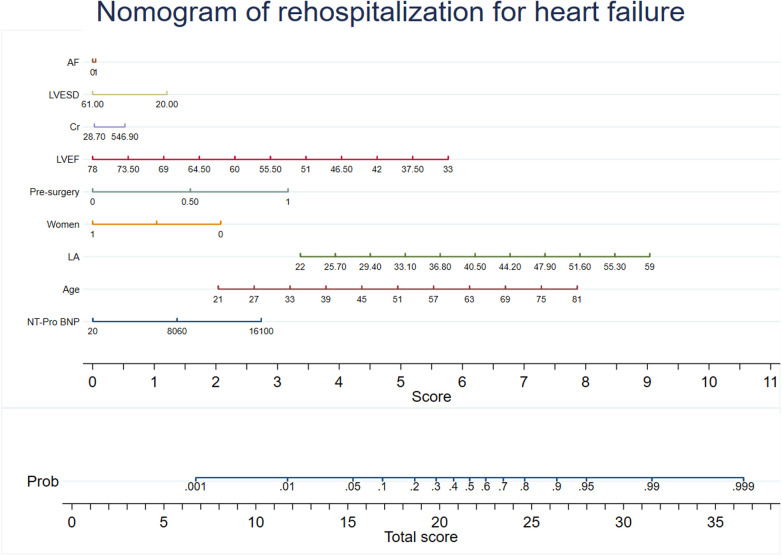
Nomogram of rehospitalization for heart failure. The nomogram was constructed based on the regression coefficient in the model.

Predictive models were developed using XG Boost to evaluate mortality and rehospitalization for heart failure. The predictive variables for the mortality risk included serum creatinine (Cr), NT-Pro BNP, C-reactive protein (CRP), left atrial size (LA), cardiac troponin I (TNI), age, aortic cross clamp time, left-ventricular ejection fraction (LVEF), and left-ventricular end-diastolic volume (LVESD). The predictive variables for the risk of rehospitalization for heart failure included NT-Pro BNP, age, LA, sex, previous surgical procedure, LVEF, Cr, LVESD, and atrial fibrillation (AF). Coefficients and the OR values of the prediction system are shown in Supplementary Tables S3, S4. The recommended variables consistent with the mortality risk prediction model were age, LVEF, LVESD, NT-Pro BNP, LA, aortic cross clamp time, and Cr. Nomograms for the risk prediction system were developed based on the regression coefficients, which enhances its clinical applicability (Figures 2, 3).

**Figure 4:**
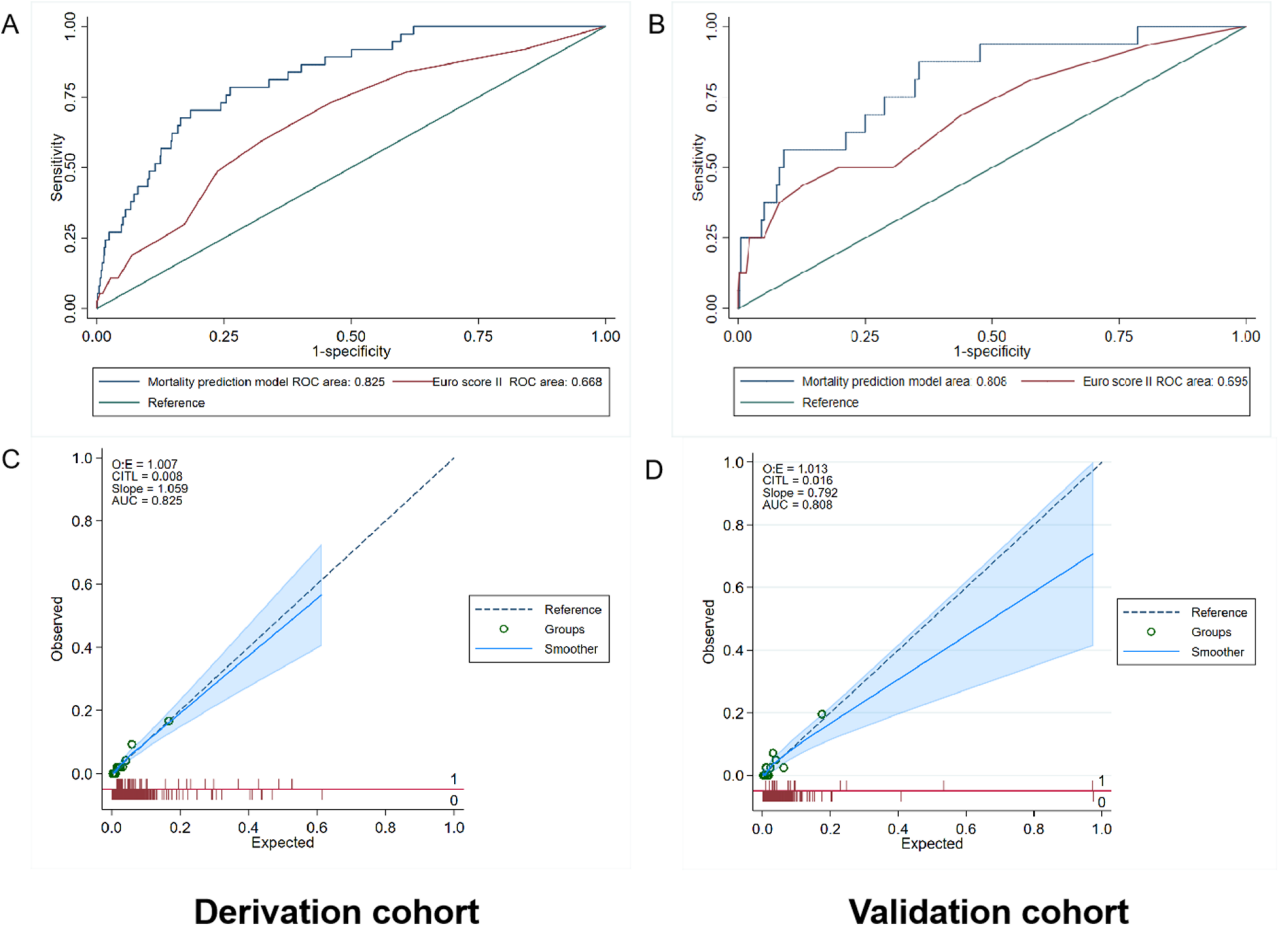
Performance evaluation in terms of mortality prediction model in the derivation and validation cohorts. **(A)** The area under the receiver operating characteristic curve (AUC) of the mortality prediction model in the derivation cohort was 0.825 [95% confidence interval (CI): 0.764–0.886], which was better than that of Euro score II (*p* < 0.001). **(B)** The AUC of the mortality prediction model in the external validation cohort was 0.808 (95% CI: 0.699–0.917), which was also better than that of Euro score II (*p* < 0.001). **(C,D)** Calibration plot of the mortality prediction model in the derivation and validation cohort. The dashed diagonal line represents perfect calibration. The *x*-axis is the predicted probability estimated by the model and the *y*-axis is the actual probability.

The AUC values of the mortality prediction model in the derivation and validation cohorts were 0.825 (95% CI: 0.764–0.886) and 0.808 (95% CI: 0.699–0.917), respectively (Figure 4). The AUC of the death prediction model was higher than that of the Euro score II in the derivation cohort and the validation cohort (*p* < 0.001). The mortality prediction model significantly increased NRI and IDI compared with Euro score II in the derivation cohort and the validation cohort (*p* < 0.05) (Tables 2, 3).

**Table 2:** Improved model performance over the euro SCORE II for mortality in the derivation cohort.

Statistic	Estimate	95% CI		*p* value	Statistic	Estimate	95% CI		*p* value
NRI (Controls)	−0.100	−0.144	−0.056	0	IDI (Controls)	2.885	2.715	3.054	0
NRI (Cases)	0.378	0.130	0.627	0.003	IDI (Cases)	−4.605	−5.719	−3.490	0
NRI (Overall)	0.278	0.026	0.531	0.031	IDI (Overall)	−1.720	−2.848	−0.593	0.003

CI, confidence interval; NRI, net reclassification improvement; IDI, integrated discrimination improvement. Performance improvement compared with Euro SCORE II.

**Table 3:** Improved model performance over the euro SCORE II for mortality in the validation cohort.

Statistic	Estimate	95% CI		*p* value	Statistic	Estimate	95% CI		*p* value
NRI (Controls)	−0.416	0.501	0.331	0	IDI (Controls)	2.609	2.375	2.844	0
NRI (Cases)	1.000	0.510	1.490	0	IDI (Cases)	−5.025	6.906	3.144	0
NRI (Overall)	0.584	0.087	1.082	0.021	IDI (Overall)	−2.415	4.311	0.520	0.013

CI, confidence interval; NRI, net reclassification improvement; IDI, integrated discrimination improvement. Performance improvement compared with Euro SCORE II.

**Figure 5:**
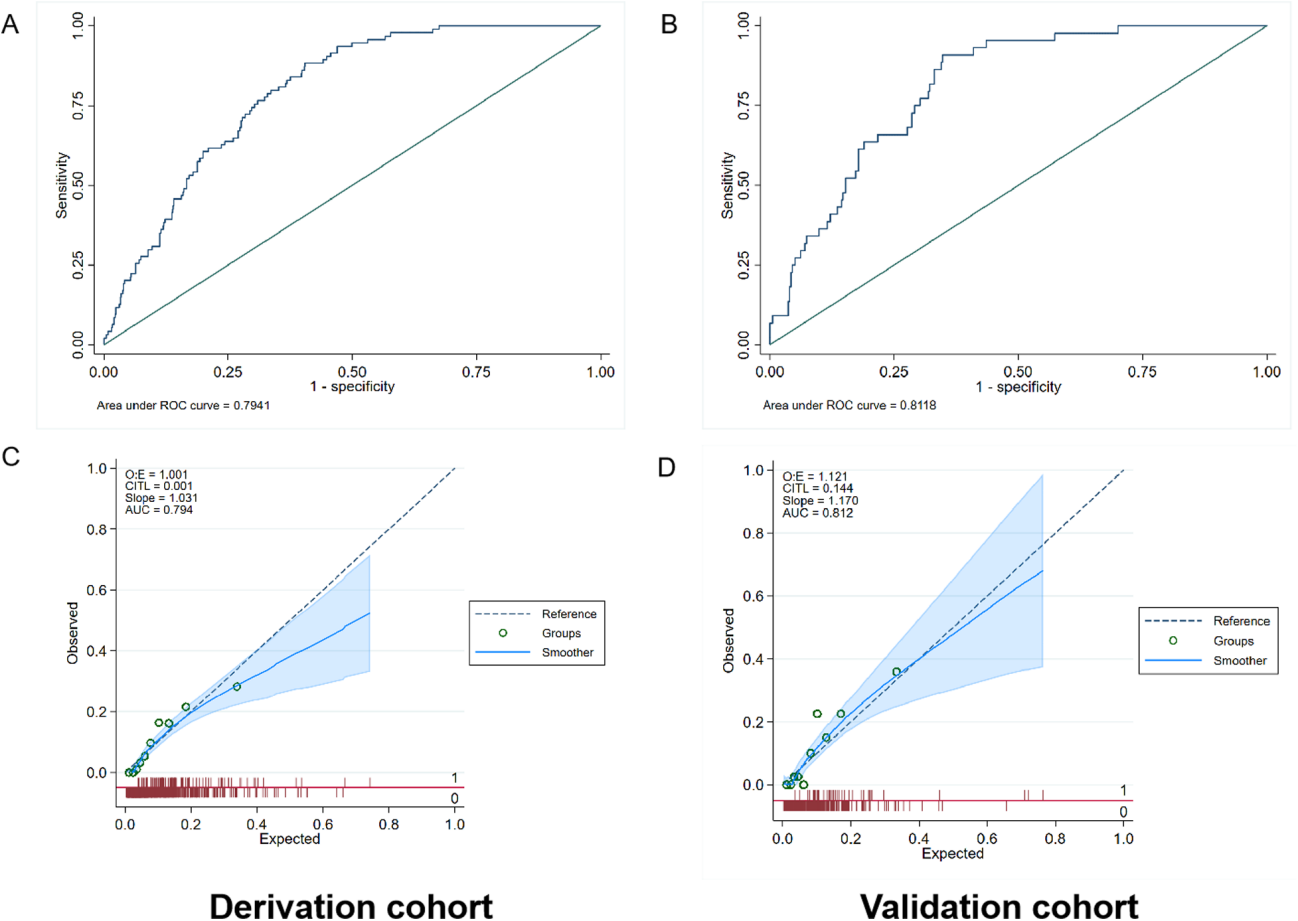
Performance evaluation in terms of rehospitalization for heart failure prediction model in the derivation and validation cohorts. **(A)** The area under the receiver operating characteristic curve (AUC) of the rehospitalization prediction model was 0.794 [95% confidence interval (CI): 0.756–0.832] in the derivation cohort. **(B)** The AUC of the rehospitalization prediction model was 0.812 (95% CI: 0.758–0.866) in the validation cohort. **(C,D)** Calibration plot of the mortality prediction model in the derivation and validation cohort. The dashed diagonal line represents perfect calibration. The *x*-axis is the predicted probability estimated by the model and the *y*-axis is the actual probability.

The AUC values of the rehospitalization for heart failure prediction model in the derivation and validation cohorts were 0.794 (95% CI: 0.756–0.832) and 0.812 (95% CI: 0.758–0.866), respectively (Figure 5). Using the Hosmer simulator chi-square test in the derivation and validation cohorts for evaluation, we found that both models exhibited sufficient calibration in both cohorts (Figures 4, 5). This indicates that the predicted probability is consistent with the actual observed results.

**Figure 6:**
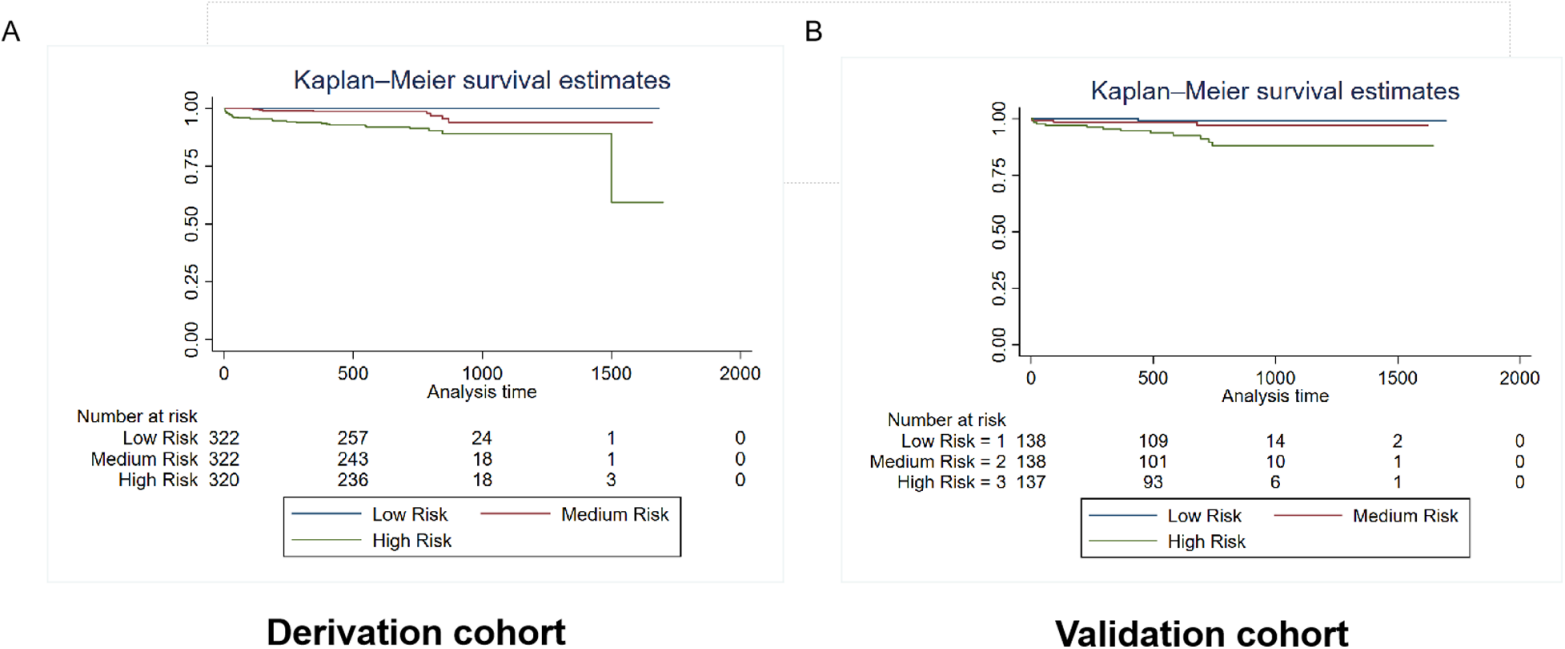
Kaplan–Meier estimates of mortality in the derivation and validation cohorts. The survival rate was observed using the Kaplan–Meier curves and compared using the log-rank test. **(A)** Kaplan–Meier estimates of the survival rate in the derivation cohort using the mortality prediction model (*p* < 0.0001). **(B)** Kaplan–Meier estimates of the survival rate in the external validation cohort using the mortality prediction model.As shown by the Kaplan–Meier curves, the two cohorts showed significant differences in mortality among the three risk-score groups (*p* < 0.0001).

**Figure 7:**
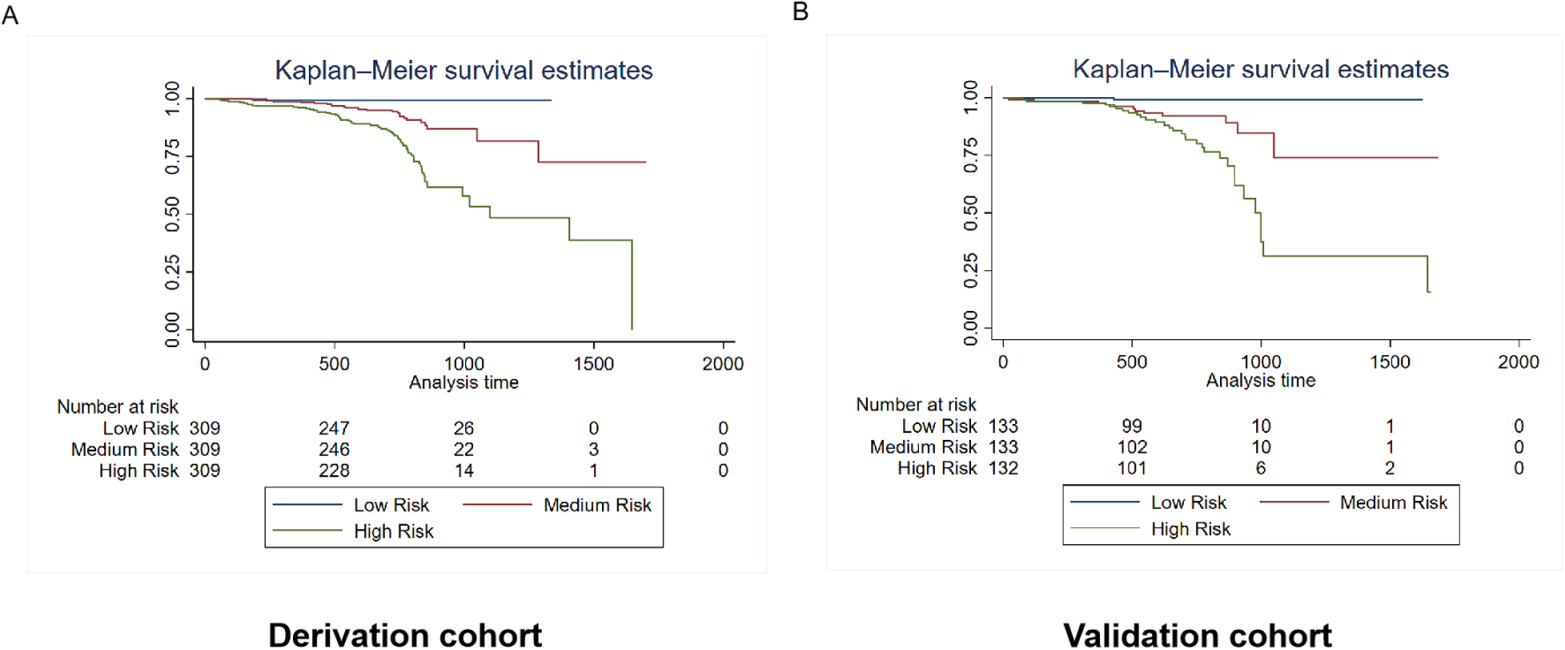
Kaplan–Meier estimates of rehospitalization for heart failure in the derivation and validation cohorts. The survival rate was observed using the Kaplan–Meier curves and compared using the log-rank test. **(A)** Kaplan–Meier estimates of rehospitalization for heart failure in the derivation cohort using the rehospitalization prediction model (*p* < 0.0001). **(B)** Kaplan–Meier estimates of rehospitalization for heart failure in the validation cohort using the rehospitalization prediction model (*p* < 0.0001). As shown by the Kaplan–Meier curves, the two cohorts showed significant differences in rehospitalization for heart failure among the three risk-score groups (*p* < 0.0001).

Patients were categorized into three risk subgroups using the quartile method. The Kaplan-Meier curves demonstrate significant differences in mortality and rehospitalization for heart failure between the derivation and validation cohorts across various risk-assessment subgroups. This indicates that the risk prediction system is capable of effectively stratifying the risk of adverse events (Figures 6, 7).

## Discussion

We developed and validated a personalized risk prediction system using data from 1,378 patients after mitral valve repair surgery to predict the risk of mortality and rehospitalization for heart failure. We identified and screened the predictive indicators from clinical data to develope and validate the risk prediction system. The predictive variables in the mortality risk model included Cr, NT-Pro BNP, CRP, LA, TNI, age, aortic cross clamp time, LVEF, and LVESD. The predictive variables in the heart failure readmission prediction model included age, NT-pro BNP, age, LA, sex, surgical history, LVEF, Cr, LVESD, and AF. We found that this risk prediction system has satisfactory discriminatory ability and risk stratification ability in predicting postoperative all-cause mortality and rehospitalization for heart failure in patients undergoing mitral valve repair surgery. In comparison to Euro score II, the mortality risk prediction model demonstrates an enhanced ability to differentiate and forecast all-cause mortality.

We used to develop and validate a risk model for the overall population of patients undergoing mitral valve surgery. However, compared to mitral valve replacement surgery, mitral valve repair is more effective in improving patient prognosis and has been shown to reduce mortality. Nevertheless, after correcting hemodynamics post-surgery, the variation in the degree of myocardial fibrosis may necessitate a period for patients to recover their myocardial contractility and achieve restoration of ventricular volume, the restoration of cardiac function and improvement in quality of life remain critical concerns for both physicians and patients. This study primarily focuses on a more concentrated population undergoing mitral valve repair surgery, examining outcomes related to mortality and readmission due to heart failure. The aim is to conduct a comprehensive assessment of risks faced by patients following discharge. Based on the various types of variables within the risk model, a comprehensive assessment of patients is conducted. For high-risk patients, regular monitoring and follow-up will be implemented concerning modifiable risk factors in order to strive for better prognostic outcomes.

In this case, predictive models can help identify high-risk patients for death and readmission and guide direct and specific interventions for those who may benefit the most by identifying key risk factors. Machine learning enables artificial intelligence to learn complex rules and recognize patterns from multidimensional datasets, which has been effectively applied in many fields of cardiology, such as precise phenotype, diagnosis, and prognosis, including predicting readmission and mortality rates (8, 11). It is a new method that meets the urgent requirements of personalized risk assessment, and it has significant advantages in clinical outcome prediction and risk factor assessment. XG Boost is widely used due to its advantages of short training time and high accuracy (12). We have illustrated that machine learning can be utilized to assist in postoperative risk prediction by capturing complex and advanced interactions.

The variables included in both the mortality and the rehospitalization for heart failure prediction models are age, Cr, NT-pro BNP, LA, LVEF, LVESD, and aortic cross clamp time. As age increases, physiological reserves decrease significantly at the level of myocardial organs, and patients are more prone to myocardial injury, leading to an increase in left-ventricular volume load and heart failure. Older patients are relatively frail and may experience decompensation at lower thresholds, requiring more frequent hospitalization treatment (13). Therefore, it is crucial to identify elderly patients who are prone to adverse events (14). Cardiovascular and chronic kidney diseases often coexist and share common risk factors in their pathological and physiological development. They can worsen each other's prognosis, and impaired kidney function is one of the strongest predictors of heart failure and poor prognosis (15). NT-proBNP is an endogenous cardiac hormone mainly released by ventricular muscle cells in response to the stretching of myocardial cells (16). NT-proBNP is of great clinical practical value as a biomarker to assist in diagnosis and treatment of patients with heart failure. Although this indicator is not included in the commonly used mortality prediction model, the guidelines also recommend the use of NTproBNP in the valve management process (3). LVEF is an important clinical indicator for measuring myocardial contractile function. Left-ventricular size parameters, such as LVESD, largely reflect the left-ventricular remodeling of patients. Patients with larger left ventricles are at a heightened risk of adverse events resulting from subsequent deterioration of LVEF. LVEF and LVESD reflect the left-ventricular function status of patients and have important prognostic value and are widely used in risk assessment and management of many cardiovascular diseases (3, 17).

The size of the left atrium is a predictor of poor prognosis. It may be the result of left-ventricular systolic and diastolic dysfunction, as well as activation of neurohormones and inflammation, leading to heart failure and adverse events (18). The aortic cross clamp time has a significant impact on myocardial injury in surgical patients, possibly because prolonged aortic occlusion leads to myocardial ischemia–reperfusion, which can affect the recovery and prognosis of myocardial function. Therefore, controlling the aortic cross clamp time during surgery is crucial (19).

The remaining indicators in the mortality risk prediction model are TNI and CRP. TNI is an indicator for evaluating myocardial injury and has significant implications for the risk of death after cardiac surgery (20). CRP is a sensitive indicator that reflects inflammation in the body and is an indicator of systemic inflammation. It has been widely accepted as an effective risk indicator that can predict cardiovascular events and has prognostic value for major cardiovascular mortality (21). The indicators of the rehospitalization prediction model that are not included in the mortality risk prediction model are sex and AF. Women often present later in the progression of their disease and exhibit poorer preoperative baseline risk profiles compared to males. Additionally, female anatomy is reported to pose greater surgical challenges due to smaller, more tortuous coronary arteries and reduced diameter cardiac valves. Women are considered a risk factor for mortality in cardiac surgery EuroSCORE II risk score. However, the risk of rehospitalization for heart failure in women may be lower, potentially due to the female heart appears to respond to injury in a manner distinct from that of the male heart. For instance, studies have indicated that women exhibit less ventricular remodeling, maintain better right ventricular function, and possess greater protection against ventricular arrhythmias, neurohormonal activation, genetic mutations, myocytenecrosis and apoptosis. Some of these advantages may be attributed to factors associated with pregnancy and sex-specific differences in gene expression (22, 23). The clinical outcomes of women with heart failure are better than those of men (24). AF is significantly associated with the incidence of heart failure, and both often coexist, with an increased risk of adverse events. Control of AF can improve heart failure outcomes (25, 26).

Furthermore, when incorporating variables, we also considered the right cardiac function indicator TAPSE (Tricuspid annular plane systolic excusion), which may influence patient prognosis. The reason this variable is not included in the risk system is probably because, the majority of patients in our overall study population had no significant reduction in ejection fraction and did not show significant deterioration of right heart function at the onset of heart failure symptoms or detection of mitral regurgitation.This indicator may possess enhanced predictive value for adverse events in patients with reduced ejection fraction (27).

In summary, the predictive indicators included in this risk prediction system reflect the comprehensive pathological status of the individual, explaining the model's good discrimination ability and satisfactory risk stratification ability.

## Strength

The main advantage of our research is that our developed risk prediction assessment aims to investigate the comprehensive outcomes of death and rehospitalization for heart failure, using commonly used predictor variables to predict the prognosis of patients undergoing mitral valve repair. By accurately predicting cardiovascular events, it is possible to reduce the risk of adverse cardiovascular events through continuous monitoring and management of key indicators of the risk prediction system.

## Limitation

First, the data utilized in this research originated from a single center; Therefore, the applicability of the system still needs further validation. Nevertheless, the study had a relatively large total sample size of patients and the population was representative. We will also collaborate further with other centers based on this, establishing follow-up survey questionnaires to obtain more reliable results for model enhancement. Second, the weights of risk indicators in the system may change over time, requiring real-time updates of the database and algorithm upgrades. Third, our system requires further integration with the healthcare system and develop related software to eliminate manual calculation processes and promote the further use of the system in clinical practice.

## Conclusions

We developed and validated a risk prediction system for patients undergoing mitral valve repair surgery. It has satisfactory discriminatory ability and risk stratification ability in predicting all-cause mortality and rehospitalization for heart failure. The machine learning approach is not only feasible and effective, but also have the potential to significantly impact the optimization of medical management.

## Data Availability

The raw data supporting the conclusions of this article will be made available by the authors, without undue reservation.
