# Supplementary material for: Personalized risk prediction of mortality and rehospitalization for heart failure in patients undergoing mitral valve repair surgery
Source: Front Cardiovasc Med. 2024 Nov 1;11:1470987. doi: 10.3389/fcvm.2024.1470987 (PMC11563966; doi:10.3389/fcvm.2024.1470987)
Supplement: Supplementary file 1 [file Supplementarymaterial.pdf]

Supplementary Material

Supplementary Methods 1.

1 Candidate variables and definitions

2 (1) Demographic variables: sex, age, Euro Score II

3 (2) Clinical variables: smoking history, drinking history, hypertension, diabetes, hyperlipidemia, coronary heart disease(CAD), syncope, atrial fibrillation(AF), previous myocardial infarction(Pre-MI), previous surgery(Pre-surgery), previous valve surgery(Pre-valvesurgery), infective endocarditis, previous central nervous system disease(Centralnervous) and peripheral vascular disease.

4 (3) Imaging variables: left atrial (LA) anterior posterior diameter, ventricular septal thickness (VST), left ventricular end diastolic volume (LVEDD), left ventricular wall thickness (Lv thickness), left ventricular ejection fraction (LVEF), and cross valve pressure gradient(SPAP).

5 (4) Laboratory variables: creatine kinase MB (CKMB), cardiac troponin I (TnI), C-reactive protein (CRP), serum creatinine (Cr), hemoglobin (Hb), lymphocyte count (Lym), neutrophil count (Neu), platelet (PLT), and N-terminal pro-brain natriuretic peptide (NT-Pro BNP).

6 (5) Surgical related variables included combined aortic surgery, combined aortic valve surgery(avr), combined tricuspid valve repair surgery (tvp), combined radiofrequency ablation (ra), combined coronary artery bypass grafting (cabg), combined ventricular septal repair (vsd), combined atrial septal repair (asd), and aortic cross clamp time.

Supplementary Figure 1. Flow chart for inclusion and exclusion of patients.

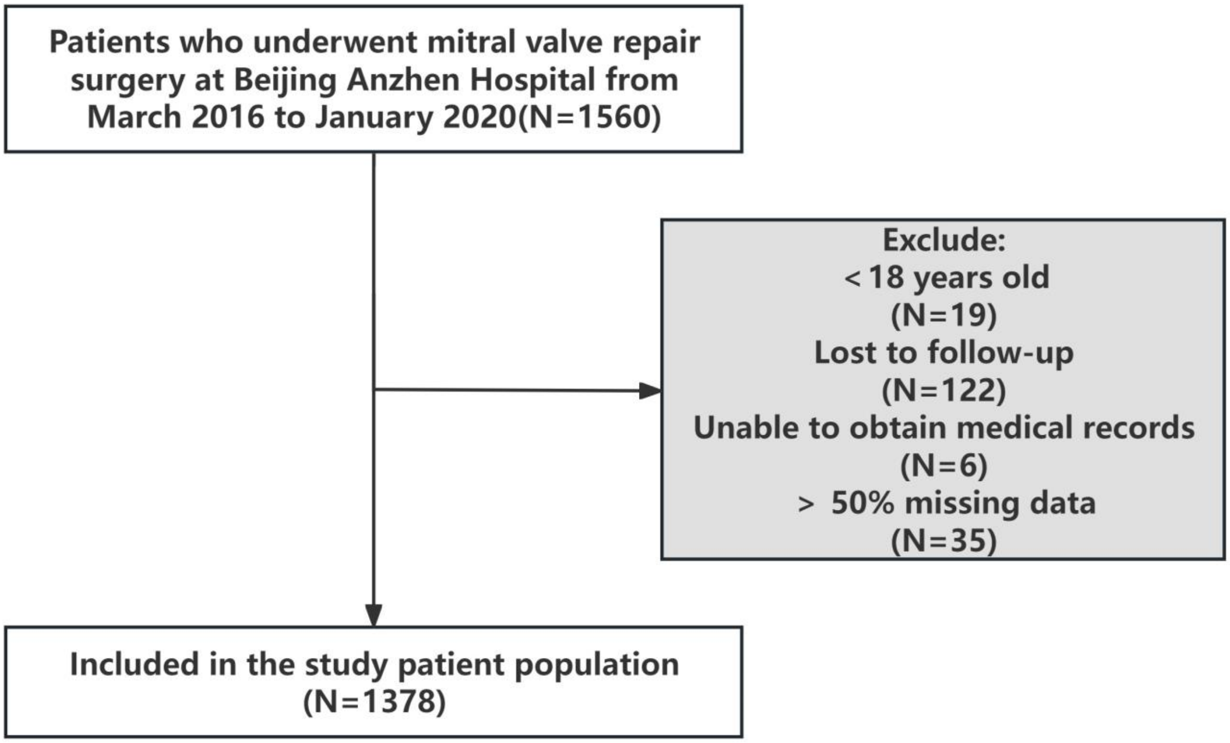

Supplementary Figure 2. Comparative Analysis of Performance between XGBoost and Traditional Logistic Regression

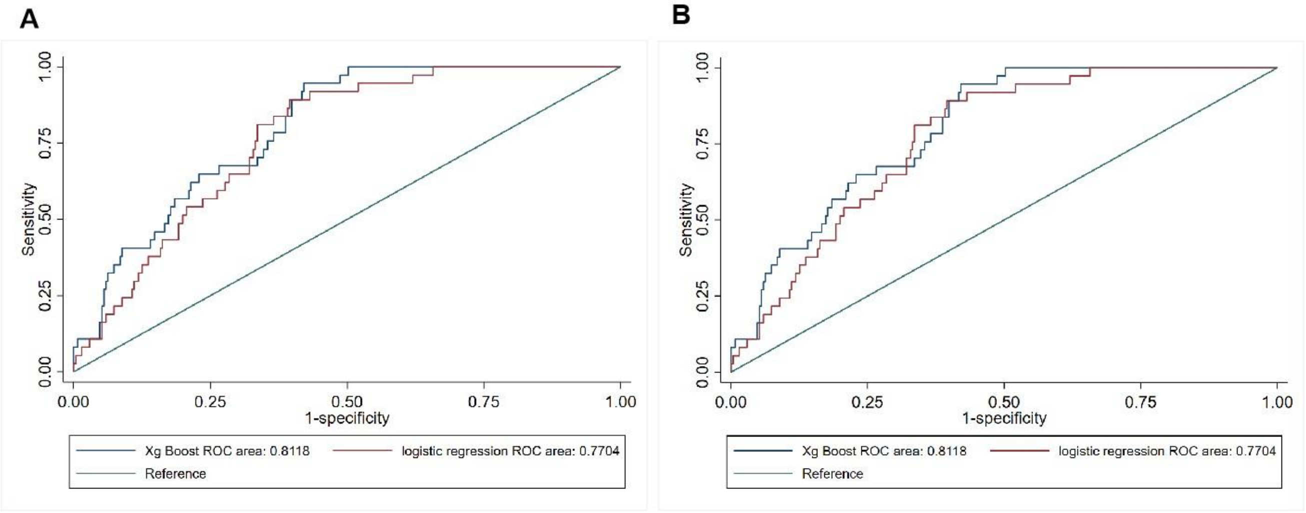

(A) Comparative analysis of the efficacy of xgboost and traditional logistic regression in developing mortality risk models.

(B) Comparative analysis of the efficacy of xgboost and traditional logistic regression in developing rehospitalization for heart failure models.

Supplementary Figure 3. Radar Chart of Risk Models

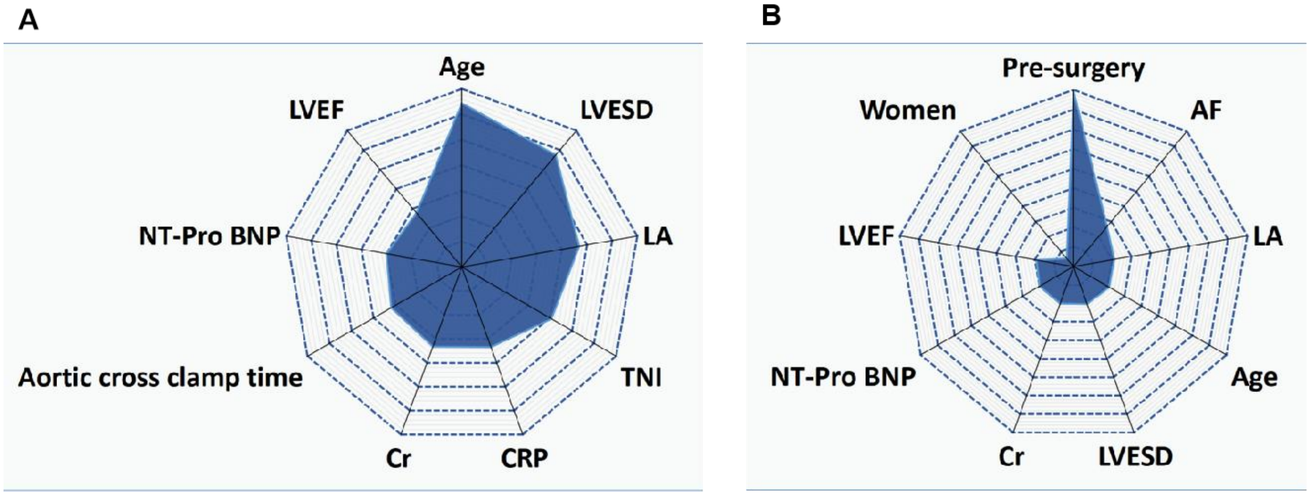

(A) Radar Chart of mortality risk model.

(B) Radar Chart of rehospitalization for heart failure model.

Supplementary Figure 4. Radar Chart of hyperlipidemia, history of central nervous system diseases, and the level of creatine kinase MB for mortality prediction model

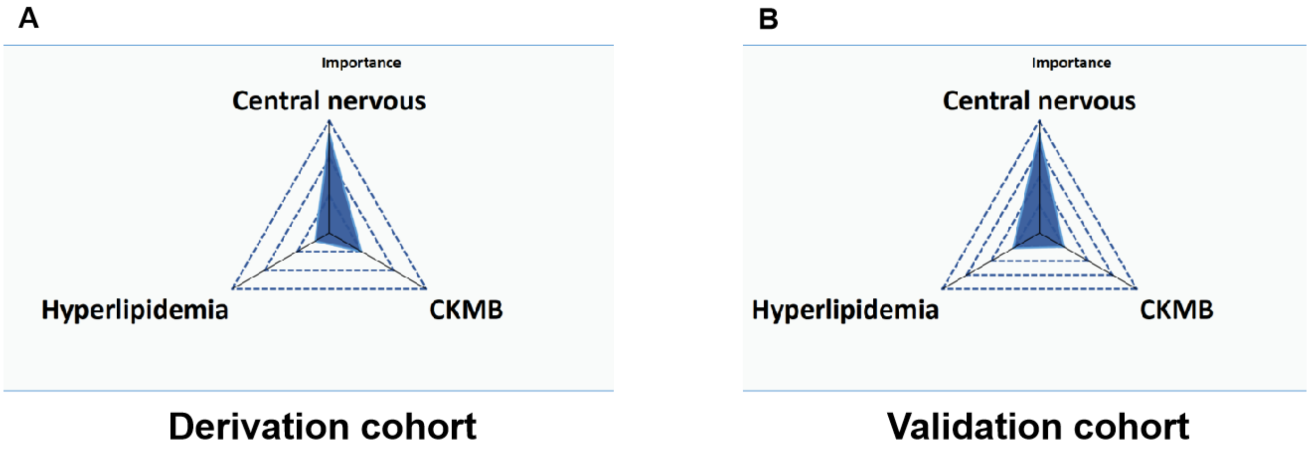

(A) Radar Chart of hyperlipidemia, history of central nervous system diseases, and the level of creatine kinase MB for mortality prediction in derivation cohort.

(B) Radar Chart of hyperlipidemia, history of central nervous system diseases, and the level of creatine kinase MB for mortality prediction in validation cohort.

Supplementary Figure 5. Performance evaluation in terms of rehospitalization for heart failure prediction model according to NT-Pro BNP levels.

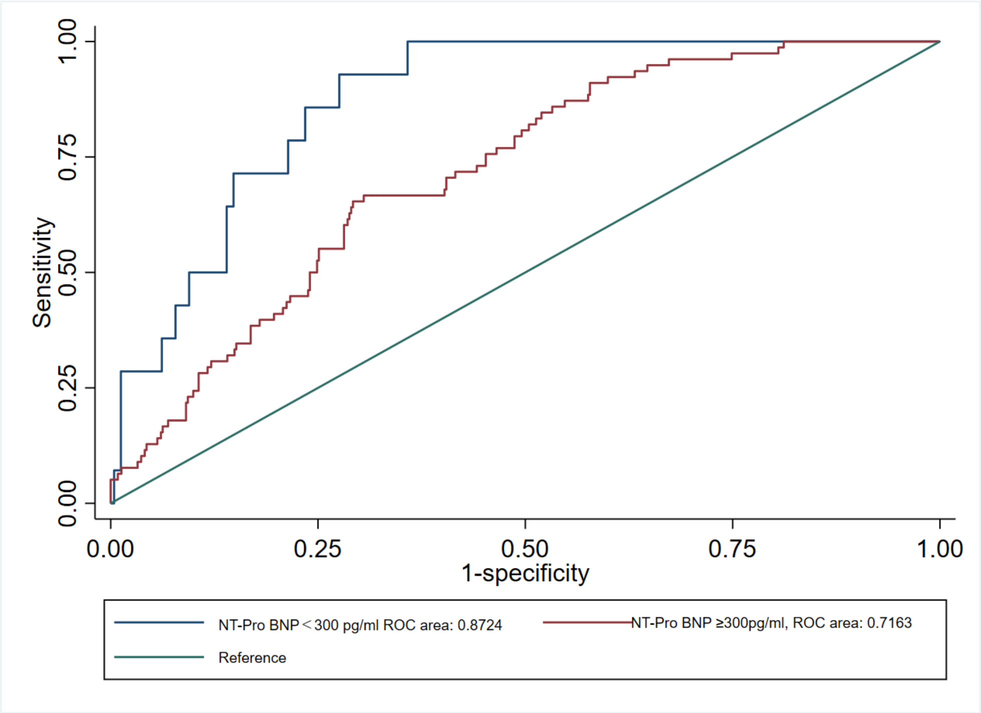

Supplementary Figure 6. Calibration plot of the Euro score II for predicting mortality

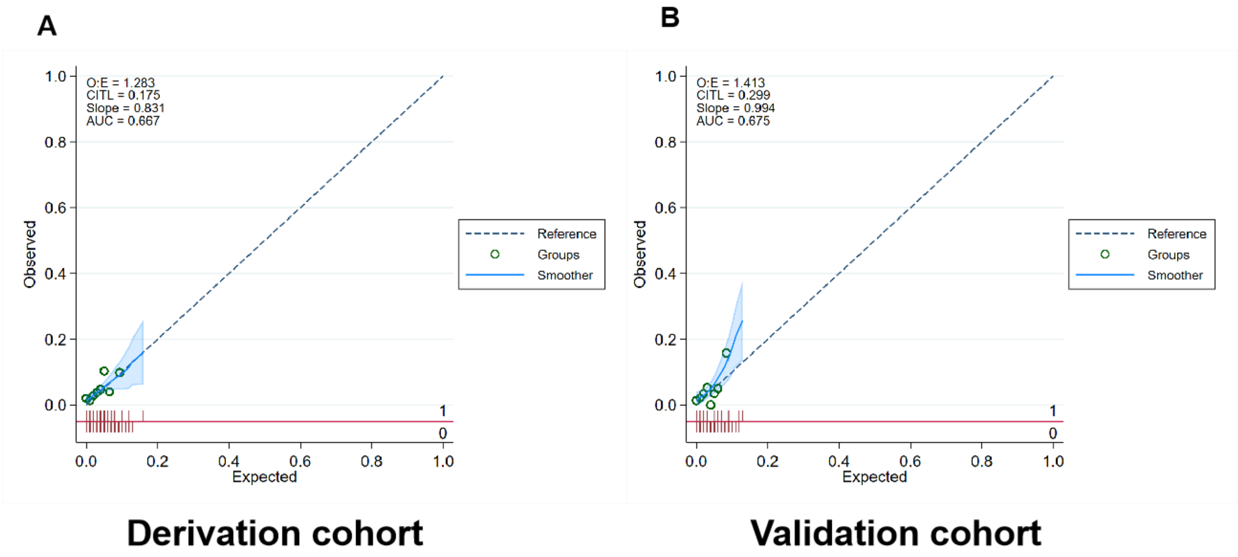

(A) Calibration plot of the Euro score II for predicting mortality in the derivation cohort.

(B) Calibration plot of the Euro score II for predicting mortality in the validation cohort.

Supplementary Figure 7. Correlation heat map of predictor variables of risk prediction models

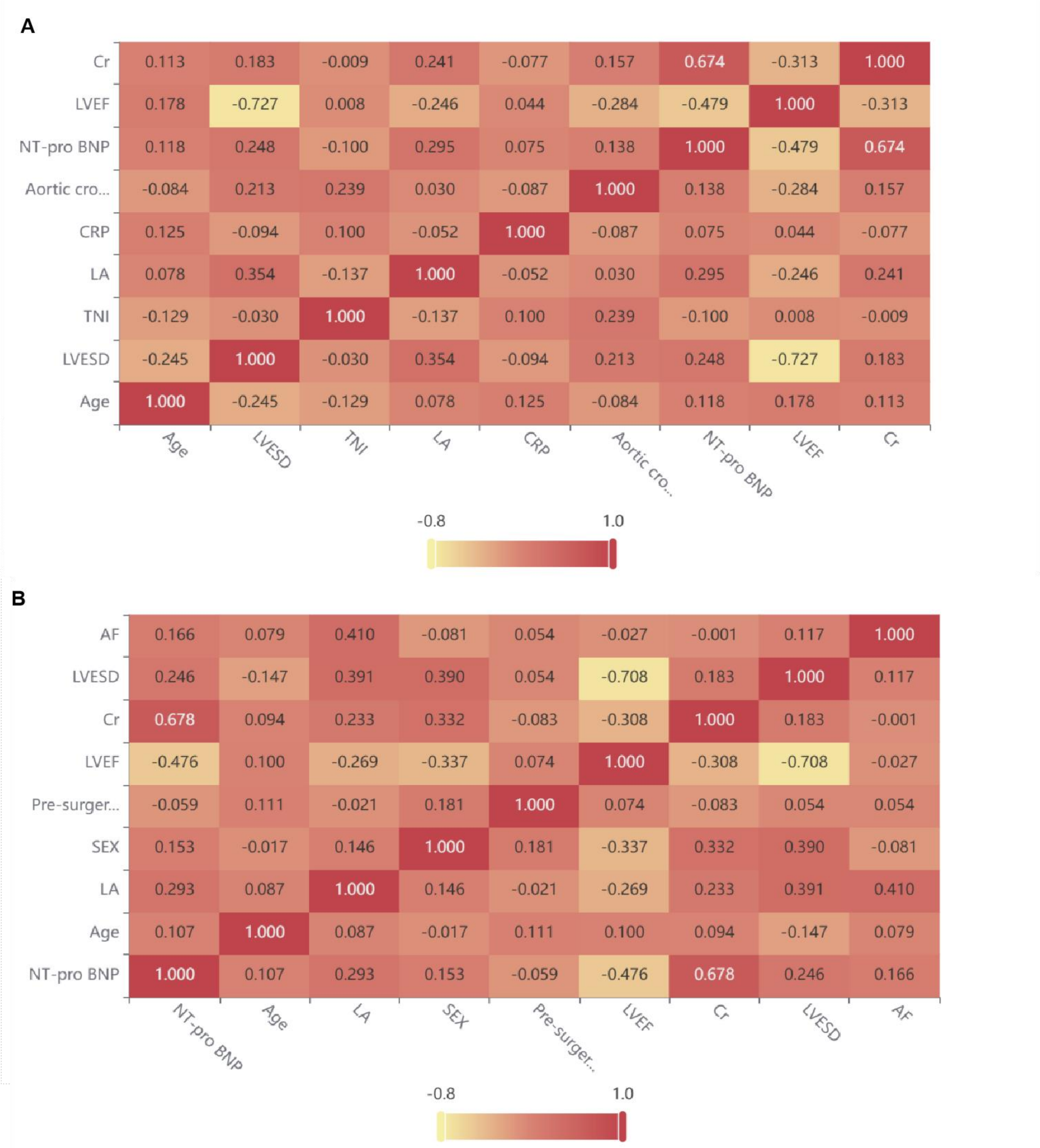

(A) Correlation heat map of predictor variables of mortality risk prediction model

(B) Correlation heat map of predictor variables of rehospitalization for heart failure risk prediction model

Supplementary Figure 8. Performance evaluation of the days of diagnosed with heart failure on the risk of mortality and rehospitalization for heart failure.

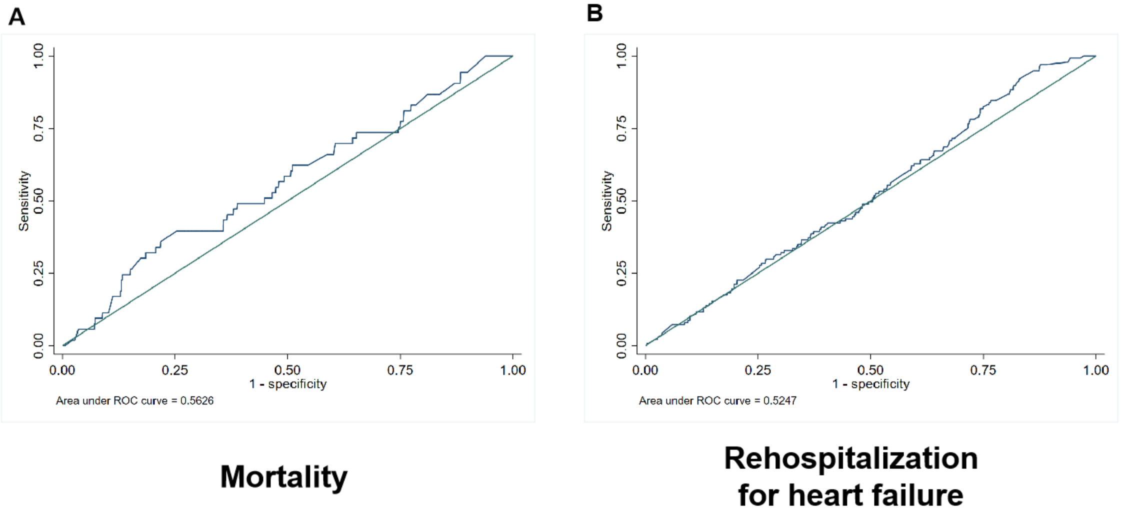

(A) The area under the receiver operating characteristic curve (AUC) of the days of diagnosed with heart failure for predicting mortality in the overall population.

(B) The area under the receiver operating characteristic curve (AUC) of the days of diagnosed with heart failure for predicting rehospitalization for heart failure in the overall population.

Supplementary Figure 9. Performance evaluation of NYHA class on the risk of mortality and rehospitalization for heart failure.

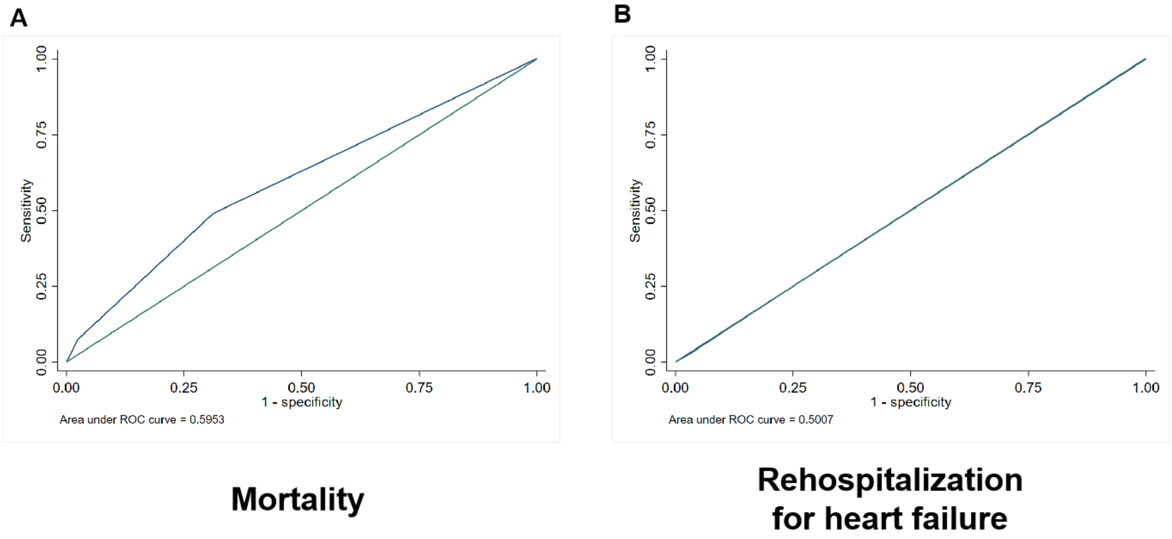

(A) The area under the receiver operating characteristic curve (AUC) of NYHA class for predicting mortality in the overall population.

(B) The area under the receiver operating characteristic curve (AUC) of NYHA class for predicting rehospitalization for heart failure in the overall population.

Supplementary Table 1. Missing value for mortality

Variables	Missing	Total	Percent
**Aortic cross clamp time**	25	1,378	1.81
**NT-Pro Bnp**	427	1,378	30.99
**CKMB**	62	1,378	4.5
**TNI**	67	1,378	4.86
**Hb**	70	1,378	5.08
**Lym**	38	1,378	2.76
**Neu**	38	1,378	2.76
**Cr**	37	1,378	2.69
**PLT**	71	1,378	5.15
**LA**	183	1,378	13.28
**VST**	278	1,378	20.17
**LVEDD**	107	1,378	7.76
**LVESD**	190	1,378	13.79
**Lv thicknes**	285	1,378	20.68
**LVEF**	112	1,378	8.13

Supplementary Table 2. Missing values for re-hospitalization

Variables	Missing	Total	Percent
**Aortic cross clamp time**	23	1,325	1.74
**NT-Pro Bnp**	417	1,325	31.47
**CKMB**	60	1,325	4.53
**TNI**	64	1,325	4.83
**Hb**	66	1,325	4.98
**Lym**	35	1,325	2.64
**Neu**	35	1,325	2.64
**Cr**	35	1,325	2.64
**PLT**	67	1,325	5.06
**LA**	164	1,325	12.38
**VST**	249	1,325	18.79
**LVEDD**	97	1,325	7.32
**LVESD**	175	1,325	13.21
**Lv thickness**	256	1,325	19.32
**LVEF**	106	1,325	8
**CRP**	286	1,325	21.58

Supplementary Table 3 Baseline features of included cohorts (n = 1325) *

Variables	derived cohort (n=927)	validation cohor (n=398)	P Value
**Demographic variables**			
Women (N,%)	530 (57.17)	220(55.28)	0.52
Age (Median±Sd)	56.55± 11.58	55.47± 11.70	0.12
Euro scoreII	2.85±2.57	2.82±2.61	0.86
**Clinical variables**			
Smoke (N,%)	224（24.16）	93（23.37）	0.76
Drinking (N,%)	151（16.29）	73（18.34）	0.36
Hypertension (N,%)	307（33.12）	123（30.90）	0.43
Diabetes (N,%)	77（8.31）	32（8.04）	0.87
Hyperlipidemia (N,%)	94（10.14）	30（7.54）	0.14
CAD (N,%)	152（16.40）	68（17.09）	0.76
Syncope (N,%)	17（1.83）	8（2.01）	0.83
AF (N,%)	329（35.49）	145（36.43）	0.74
Pre-MI (N,%)	25（2.70）	7（1.76）	0.31
Pre-surgery (N,%)	35（3.78）	15（3.77）	0.99
Pre-valvesurgery (N,%)	8（0.86）	4（1.01）	0.80
Renal insufficiency (N,%)	14（1.51）	3（0.75）	0.26
Infect endocarditis (N,%)	15（1.62）	11（2.76）	0.17
Central nervous (N,%)	52（5.61）	31（7.79）	0.13
Lung disease (N,%)	19（2.05）	8（2.01）	0.96
Peripheral vd (N,%)	10（1.08）	3（0.75）	0.58
**Imaging variables**			
LA (Median±Sd)	38.52±6.50	38.59±6.80	0.86
VST (Median±Sd)	10.09±1.79	9.98±1.85	0.38
LVEDD (Median±Sd)	47.61±5.94	47.70±5.96	0.80
LVESD (Median±Sd)	32.88±6.03	32.78±6.21	0.81
Lv thicknes (Median±Sd)	9.76±1.49	9.63±1.40	0.20
LVEF (Median±Sd)	57.33±7.62	57.85±7.64	0.28
SPAP (Median±Sd)	23.67±8.04	23.68±7.97	0.98
**Laboratory variables**			
NT-Pro BNP(Median±Sd)	916.56±1553.97	1223.974±3047.21	0.05
CKMB (Median±Sd)	54.23± 42.14	55.06±42.55	0.75
TNI (Median±Sd)	5.10±6.08	4.79±7.49	0.45
CRP (Median±Sd)	25.42±40.38	26.99±43.10	0.57
Cr (Median±Sd)	72.00±30.45	70.16±32.55	0.33

Hb (Median±Sd)	103.51±20.95	103.62±21.28	0.94
Lym (Median±Sd)	1.39±1.02	1.39±0.97	0.96
Neu (Median±Sd)	9.98±5.18	10.20±7.28	0.53
PLT (Median±Sd)	151.19±70.79	150.29±73.36	0.84
**Surgical variables**			
Combined aortic surgery (N,%)	28（3.02）	10（2.51）	0.61
Combined avr (N,%)	109（11.76）	49（12.31）	0.78
Combined tvp (N,%)	520（56.09）	220（55.28）	0.78
Combined cabg (N,%)	115（12.41）	55（13.82）	0.48
Combined ra (N,%)	271（29.23）	120（30.15）	0.74
Combined asd (N,%)	20（2.16）	8（2.01）	0.86
Combined vsd (N,%)	4（0.43）	1（0.25）	0.62
Aortic cross clamp time (Median±Sd)	93.33±40.82	96.74±56.12	0.22
**Follow up events**			
Days (N,%)	699.65±227.70	694.26±232.27	0.69
Re hospitalization for heart failure (N,%)	101（10.47）	37（8.96）	0.39

*For continuous variables, non-normally distributed variables are expressed as median (interquartile ranges [IQRs]) and normally distributed variables are expressed as means (standard deviation [SD]). Categorical variables are expressed in N (%). $P < 0.05$ was considered to be statistically significant.; CAD, coronary heart disease; AF, atrial fibrillation; Pre-MI, previous myocardial infarction;Pre-surgery, previous surgery; Pre-valve surg, previous valve surgery; Central nervous, previous central nervous system disease; LA, left atrial; VST, ventricular septal thickness; LVEDD, left ventricular end diastolic volume;LVESD, left ventricular end-systolic diameter ; Lv thickness, left ventricular wall thickness; LVEF, left ventricular ejection fraction; SPAP, Systolic Pulmonary Artery Pressure;NT-Pro BNP, N-terminal pro-brain natriuretic peptide ; CKMB, creatine kinase MB; TNI, cardiac troponin I; CRP, C-reactive protein; Cr, serum creatinine; Hb, hemoglobin; Lym, lymphocyte count; Neu, neutrophil count; PLT, platelet count; avr, aortic valve surgery; tvp, tricuspid valvue repair surgery; ra, radiofrequency ablation; cabg, coronary artery bypass grafting; asd, atrial septal repair; vsd, ventricular septal repair;

Supplementary Table 4. Model coefficients of mortality prediction model

Covariate	Coefficient	OR (95% CI)
Cr	0.0065715	1.006593（0.999041-1.014202）
NT-Pro BNP	0.0000998	1.0001（1.000003-1.000197）
CRP	0.0091962	1.006949（ 1.000623-1.013315）
LA	0.0330596	1.033612（0.9764422-1.094129）
TNI	0.0211782	1.021404（0.9968768-1.046535）
Age	0.0664865	1.068747（1.024995-1.114366）
Aortic cross clamp time	0.0040844	1.004093（0.9971353-1.011099）
LVEF	-0.0045182	0.995492（0.9346153-1.060334）
LVESD	0.053461	1.054916（0.9762785-1.139887）

Supplementary Table 5. Model coefficients of re hospitalization for heart failure prediction model

Covariate	Coefficient	OR (95% CI)
NT-Pro BNP	0.0000938	1.000094（0.9999491-1.000239）
Age	0.048577	1.049776（1.023611-1.07661）
LA	0.0544991	1.056011（1.015827-1.097786）
Women	0.3916278	0.2682236（0.2314977-0.662522）
Pre-surgery	1.498644	4.475617（1.865778-10.73608）
LVEF	-0.0400421	0.960749（0.919547-1.003797）
Cr	0.0018432	1.001845（0.9950528-1.008683）
LVESD	0.0154961	1.015617（0.9625726-1.071584）
AF	0.2870834	1.332535（0.8050282-2.2057）

Supplementary Table 6. Model coefficients of conventional logistic regression mortality prediction model

Covariate	Coefficient	OR (95% CI)
Renal Insufficiency	1.926485	6.865337（1.814761-25.97193）
Age	0.0578575	1.059564（1.015088-1.105989）
Aortic cross clamp time	0.0054393	1.005454（0.9985745-1.012381）
Hb	-0.0145223	0.9855827（0.9682346-1.003242）
LA	0.0229712	1.023237（0.965772-1.084121）
LVESD	0.0721642	1.074832（1.018334-1.134464）
Lv thickness	-0.2153697	0.8062433（0.5433358-1.196366）
Lym	0.2277769	1.255805（1.03777-1.51965）
NT-Pro BNP	0.0000463	1.000046（0.9999325-1.00016）
Vst	0.338514	1.402861（0.9766232-2.015127）

Supplementary Table 7. Model coefficients of conventional logistic regression re hospitalization for heart failure prediction model

Covariate	Coefficient	OR (95% CI)
Age	0.0590607	1.06084(1.03453-1.087819)
CRP	0.0000366	1.000037(0.9936293-1.006485)
Pre-surgery	1.61906	5.048344(2.185436-11.66165)
NT-Pro bnp	0.0001418	1.000142(0.9999871-1.000297)
Women	-0.8903198	0.4105244(0.2463013-0.6842445)
LVEF	-0.028852	0.9715603(0.9299193-1.015066)
Lv thickness	-0.118223	0.8884979(0.7214708-1.094193)
**LVESD**	0.0367323	1.037415(0.9830005-1.094842)

Supplementary Table 8: Model coefficients of hyperlipidemia, history of central nervous system diseases, and the level of creatine kinase MB for mortality prediction model in derivation cohort

Covariate	OR	(95% CI)
**Central nervous**	2.654797	1.000094（1.063492-6.627172）
**CKMB**	0.9987701	1.049776（0.9899478-1.007671）
**Hyperlipidemia**	0.433726	1.332535（0.1018044-1.84784）

Supplementary Table 9: Model coefficients of hyperlipidemia, history of central nervous system diseases, and the level of creatine kinase MB for mortality prediction model in validation cohort

Covariate	OR	(95% CI)
**Central nervous**	3.577388	（0.7354876-17.4003）
**CKMB**	0.9912681	（0.9766638-1.006091）
**Hyperlipidemia**	1.090383	（0.137044-8.675571）

Supplementary Table 10: Preoperative and postoperative baseline characteristics and medication of patients undergoing mitral valve repair surgery. (n = 1378) *

Variables	Derivation cohort (n = 965)	Validation cohort (n = 413)	p value
**Preoperative Baseline Characteristics**			
NYHA class			0.08
NYHA=1 (N,%)	1 (0.10)	2 (0.48)	
NYHA=2 (N,%)	638 (66.11)	297 (71.91)	
NYHA=3 (N,%)	299 (30.98)	104 (25.18)	
NYHA=4 (N,%)	27 (2.80)	10 (2.42)	
Days of diagnosed with heart failure (Median±Sd)	669.25（29.89）	602.03（35.57）	0.19
**Preoperative Medication**			
ACEI/ARB (N,%)	88（9.12）	32（7.75）	0.41
β-Blockers (N,%)	361（37.41）	147（35.59）	0.52
Digoxin (N,%)	38（3.94）	10（2.42）	0.16
Statins (N,%)	106（10.98）	34（8.23）	0.12
Diuretic (N,%)	298（30.88）	109（26.39）	0.09
Spironolactone (N,%)	107（11.09）	32（7.75）	0.06
Aspirin (N,%)	171（17.72）	68（16.46）	0.57
**Postoperative Medication**			
ACEI/ARB (N,%)	263（27.25）	111（26.88）	0.89
β-Blockers (N,%)	170（17.62）	71（17.19）	0.85
Digoxin (N,%)	233（24.15）	96（23.24）	0.71
Statins (N,%)	114（11.81）	44（10.65）	0.54
Diuretic (N,%)	847（87.78）	380（92.01）	0.02
Spironolactone (N,%)	292（30.26）	119（28.81）	0.59
Aspirin (N,%)	120（12.44）	43（10.41）	0.29

*For continuous variables, non-normally distributed variables are expressed as median (interquartile ranges [IQRs]) and normally distributed variables are expressed as means (standard deviation [SD]). Categorical variables are expressed in N (%). $P < 0.05$ was considered to be statistically significant.; NYHA, New York Heart Association classification;
